# Electropolymerization of s-Triazines and Their Charge Storage Performance in Aqueous Acidic Electrolytes

**DOI:** 10.3390/polym16233266

**Published:** 2024-11-24

**Authors:** Shaotong Pei, Bo Lan, Xueting Bai, Yunpeng Liu, Xinyang Li, Chao Wang

**Affiliations:** 1 Hebei Provincial Key Laboratory of Power Transmission Equipment Security Defense, North China Electric Power University, Baoding 071003, China; rambo596596@163.com (B.L.); xt_baii@163.com (X.B.); liuyunpeng@ncepu.edu.cn (Y.L.); lxygerenyouxiang@163.com (X.L.); 2 Department of Chemistry and Chemical Engineering, Shaanxi University of Science and Technology, Xi’an 710021, China

**Keywords:** conductive polymer, s-triazine, aqueous electrolyte, electropolymerization

## Abstract

Designing novel π-conjugated conductive polymers with abundant redox-active groups is a viable route to achieve high charge storage performance for aqueous energy storage devices. Electropolymerization is a powerful tool to construct conductive polymers. Here, s-triazine is, for the first time, electropolymerized in an aqueous acidic solution on carbon cloth. The polytriazine-coated carbon cloth electrode (PT/CC) exhibits a granular structure, with abundant pores. The charge storage performance is investigated, and a specific capacity of 101.4 mAh $g^{-1}$ at 1 A $g^{-1}$ in 1 M $H_{2}{SO}_{4}$ is achieved. Additionally, in 1 M ZnSO_4_, a specific capacity of 50.3 mAh $g^{-1}$ at 1 A $g^{-1}$ can be achieved by the PT/CC. The PT/CC behaves as a battery-type charge storage electrode, and the amino/imino and carbonyl/hydroxyl groups contribute to the charge storage, with cation insertion and extraction. A symmetric aqueous charge storage device assembled with two PT/CC electrodes exhibits an energy density of 12.92 Wh ${kg}^{-1}$ and a power density of 250 W ${kg}^{-1}$ at 1 A $g^{-1}$. After 2500 cycles at 10 A $g^{-1}$, the device retains a specific capacity of 83.3%. This study indicates that the PT is a potential candidate material for an aqueous energy storage device.

## 1. Introduction

The development of renewable energy sources requires energy storage devices with a high energy density and power density and a long cycle life [1]. In the energy storage devices, the charge can be stored through charging the double layer, where the charges with opposite signs are aligned at the electrode–electrolyte interface, and through the redox reactions of the electrode material [2]. Conducting polymers are characteristically flexible, environmentally benign, and light weight. These features are desirable in the electrode material for flexible energy storage devices [3,4]. The rich functional groups and conductive backbones of the conducting polymers endow them with good charge storage capabilities. Organic functional groups involving heteroatoms are potential redox active sites for charge storage, for example, the hydroxyl/carbonyl groups and amino/imino groups [5,6]. Also, a large conjugated backbone is essential for improving the electron conductivity in order to achieve high specific capacities and rate capabilities [7]. Novel conducting polymers have been constructed that exhibit promising charge storage properties. For example, 1,10-phenanthroline was oxidatively electropolymerized in an acidic solution, and the resulting polymer exhibited a specific capacity of 111.5 mAh g^−1^ (1 A g^−1^) in 1 M H_2_SO_4_ [8]. Poly(1,5-diaminoquinone) was formed by electropolymerization, and a specific capacitance of 645 mF cm^−2^ (1 mA cm^−2^) was achieved [9]. Electropolymerization of a carbonyl-modified dihydropyrazine derivative was achieved and exhibited 248 mAh g^–1^ (0.1 A g^−1^) when assembled in zinc-ion batteries [10]. Also, a donor–acceptor type polymer with a pyrene-4,5,9,10-tetraone unit and a carbazole unit was synthesized by electropolymerization, to achieve 202 mA h g^−1^ (200 mA g^−1^) in dual ion batteries [11].

1,3,5-Triazine, or s-triazine, has been extensively studied in pharmaceutical chemistry [12,13]. It exhibits good optical properties and is utilized as the core structure to construct molecules with various shapes [14,15]. Moreover, covalent triazine frameworks were first reported in 2008 and found applications in energy storage and conversion [16]. Meanwhile, triazine derivatives have been used as electrocatalysts for the nitrate reduction reaction (NO_3_RR), contributing to the development of more environmentally friendly methods of ammonia production [17]. Porous covalent triazine frameworks are used as electrode materials for supercapacitors, providing high energy and power densities, as well as CO_2_-capture capabilities to combat global warming [18]. Electropolymerization serves as a facile method to construct polymer coatings at conductive substrates [19]. The electropolymerization of substituted triazine has been reported, including amine- [20], thio- [21], and triphenylamine-substituted triazine [22]. However, direct electropolymerization of the triazine molecule has not been reported yet. The triazine molecule is electron deficient and can undergo reversible multiple redox processes. The triazine ring can bind to cations (through cation–π interaction) and anions (anion–σ interaction) and thus can potentially store abundant charges [23,24]. Additionally, the planar structure is favorable for the construction of conjugated polymers with high electron conductivity. Therefore, the electropolymerization of s-triazine is carried out in aqueous solution, and the charge storage properties of the polytriazine (PT) film are investigated. The charge storage performance of the PT in H_2_SO_4_ and in ZnSO_4_ aqueous solutions is investigated to understand the redox chemistry and charge storage performance of the PT film. Also, the charge storage mechanism is investigated. The PT formed by direct electropolymerization is simple and convenient to prepare, with a relatively low cost, which is conducive to its large-scale production and application in energy storage devices.

## 2. Experimental Section

### 2.1. Preparation of PT/CC

Carbon cloth (CC) was cut into small pieces of 1 × 2 cm^2^, cleaned with deionized water, and then calcined in a tubular furnace at 300 °C for 60 min. Using a CHI760E electrochemical workstation, Shanghai Chenhua Instrument Co., Shanghai, China, PT/CC was prepared by the electropolymerization of s-triazine on the CC in a three-electrode system [25]. The treated CC was clamped with a platinum plate electrode holder, serving as the working electrode (the area of the CC immersed in the electrolyte was 1 ${cm}^{2}$). A saturated calomel electrode (SCE) was used as the reference electrode, and a rinsed graphite rod served as the counter electrode. The electrolyte was 25 mL of 1 M $H_{2}{SO}_{4}$ + 5 mM triazine aqueous solution, in which the electropolymerization of s-triazine was carried out. A constant current of 0.01 A ${cm}^{-2}$ was applied to the working electrode for 20 min to obtain PT/CC-20 (the mass of the deposited film of PT/CP-20 was 0.4 mg ${cm}^{-2}$). In addition, electrodes with an electropolymerization duration of 10 min and 30 min at 0.01 A cm^−2^ were also prepared, and the obtained electrodes were labeled as PT/CC-10 and PT/CC-30 (the mass of the deposited film of PT/CC-10 and -30 were 0.2 mg ${cm}^{-2}$ and 0.6 mg ${cm}^{-2}$, respectively). For the electrodeposition, a typical *E*–*t* curve is shown in Figure S1. The deposited mass of PT on the CC is directly proportional to the electropolymerization duration, which indicates that the limiting condition is not reached for the electropolymerization.

### 2.2. Electrochemical Measurements

PT/CC, Suzhou Shengrnuo Technology Co., Suzhou, China, was used as the working electrode, a rinsed graphite rod as the counter electrode, and a saturated calomel electrode as the reference electrode for electrochemical measurements in a three-electrode system in 1 M H_2_SO_4_. A piece of Zn foil, Shanghai Chuxi Industrial Co., Shanghai, China, was used as the counter electrode when the electrolyte was 1 M ZnSO_4_. The electrochemical impedance spectroscopy (EIS) was recorded within a frequency range of 0.01~10^6^ Hz, with the alternating voltage amplitude set to 5 mV. Information on the instrumentation and the equations used is provided in the Supporting Information. The electrochemical experiments were repeated at least five times to ensure reproducibility. The error of all the reported electrochemical data was less than 5%.

## 3. Results and Discussion

**Figure 1:**
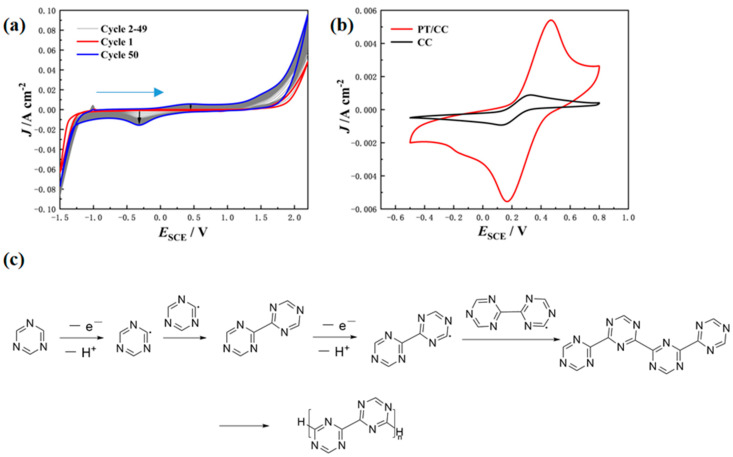
(**a**) CV of 5 mM s-triazine in 1 M $H_{2}{SO}_{4}$ at a scan rate of 100 mV $s^{-1}$ for 50 cycles (anodic first). The working electrode is CC. (**b**) The CV of a bare CC and PT/CC in 5 mM K_3_[Fe(CN)_6_] in 0.1 M KCl solution at 50 mV $s^{-1}$. (**c**) Proposed mechanism for electropolymerization of s-triazine in aqueous sulfuric acid.

The electrochemical properties of the s-triazine were firstly studied using CV in 1 M H_2_SO_4_ (Figure 1a), where the first scan was initiated from −1.5 V in the anodic direction. In the cathodic region, the main reduction initiated at −1.25 V_SCE_, which represents the irreversible reduction and further chemical reaction of the cationic radicals. A nucleation loop is seen at the cathodic region, which is frequently observed in the CV of many π-conjugated monomers and oligomers. Heinze et al. proposed that this nucleation loop originates from the comproportionation reaction between the oligomer (the coupling product of anionic radicals) and the monomer [26]. The presence of the nucleation loop indicates that the s-triazine is able to form oligomers at cathodic potentials. In literature, the strong and irreversible cathodic process is apparent for the CV of s-triazine and its derivatives, which makes the s-triazine an appealing candidate as the electron acceptor unit to construct optical active molecules. Also, the electroreduction of 1,3,5-triazine generates 4,4′-dihydrobitriazyl in acetonitrile, which is reported to hydrolyze to form tetraformamidoethane [27]. At anodic potentials, irreversible oxidation happens, which indicates that the life time of the generated cationic radical is short and undergoes further reactions. This behavior is quite similar to that of the reported electropolymerizable monomers [28]. Repetitive CV cycling is then carried out. Peaks at −1.01, −0.32, and 0.45 V_SCE_ that were not present in the first cycle were generated with cycling. This indicates the successful polymerization of the s-triazine with potential cycling. The peak at −1.01 V_SCE_ is related to the oxidation of reduced species (anionic radicals or the 4,4′-dihydrobitriazyl), and the redox peaks at 0.45 V_SCE_ and −0.4 V_SCE_ are probably related to the structures generated during electropolymerization that undergo redox reaction and participate in the redox reaction.

Figure 1b compares the CV of the PT/CC and a bare CC in a 5 mM K_3_[Fe(CN)_6_] + 0.1 M KCl solution. For the PT/CC, the redox peak separation was 304 mV, significantly higher than the theoretical value of 59 mV for the reversible 1 e^−^ transfer redox process of [Fe(CN)_6_]^3−/2−^. This indicates that the electron transfer is impeded for the PT/CC, which is caused by the presence of the polymer layer. However, the electron transfer is not completely suppressed, which indicates that there exist pinholes on the PT layer, and the deposited PT is highly porous. Furthermore, the current density of the CV of PT/CC is higher than that of the bare CC, which indicates that the number of electrochemical active sites increases. Note that the s-triazine units can be protonated in the acidic aqueous solution during electropolymerization. Both protonated and unprotonated units may attract the [Fe(CN)_6_]^3−^ that leads to higher local concentration and, therefore, higher current densities.

The electropolymerization mechanism of s-triazine is proposed as in Figure 1. Under anodic potentials, a triazine molecule is oxidized to form a radical with the loss of a proton. The generated radicals then couple with each other to form dimers. The repetitive generation of radicals with the loss of the proton forms oligomers or polymers and then precipitates at the electrode surface. Also, radicals can couple with the electron at the electrode surface to form covalent bonds with the electrode.

**Figure 2:**
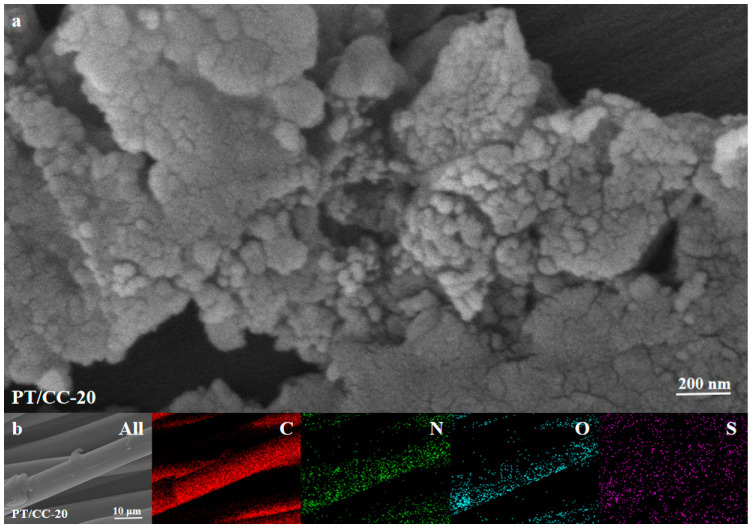
(**a**) The SEM image of PT/CC-20. (**b**) EDS elemental mapping of PT/CC-20.

Then, electropolymerization is carried out using the galvanostatic method to generate considerable amounts of PT on the CC surface. The *E*–*t* curve of electropolymerization is shown in Figure S1, and the potential of the working electrode is at ~2.0 V_SCE_. As the potential during the electropolymerization is well above the onset of the oxygen evolution reaction in acid, the electropolymerization process is accompanied by the oxygen evolution. The generated oxygen bubbles can serve as a template during polymer formation, which results in a highly porous structure. Figure 2a shows the scanning electron microscopy (SEM) image of PT/CC-20 in 1 M $H_{2}{SO}_{4}$. A rough and granular structure appears at the surface of the CC. The particulate structure has a diameter around 10 nm in size. The PT film is not compact, and pinholes and cracks exist. The cracks may be generated during electropolymerization in which oxygen bubbles evolve along with the electropolymerization process. Also, the PT film did not cover the CC substrate fully. The SEM images of PT/CC-10 and 30 are shown in Figure S6. As the electropolymerization time increases, the granular structure on the surface of the CC increases. Figure 2b shows the energy-dispersive X-ray spectroscopy (EDS) elemental mapping (C, N, S, O) of PT/CC-20. The N originates from the PT and is uniformly distributed on the CC fiber, which also indicates that the PT is generated at the CC fiber surface. The S is detected, which is due to the fact that the electropolymerization happens in sulfuric acid electrolyte, and the protonated imino groups in the PT would attract anions in the electrolyte to balance the charge of the polymer.

**Figure 3:**
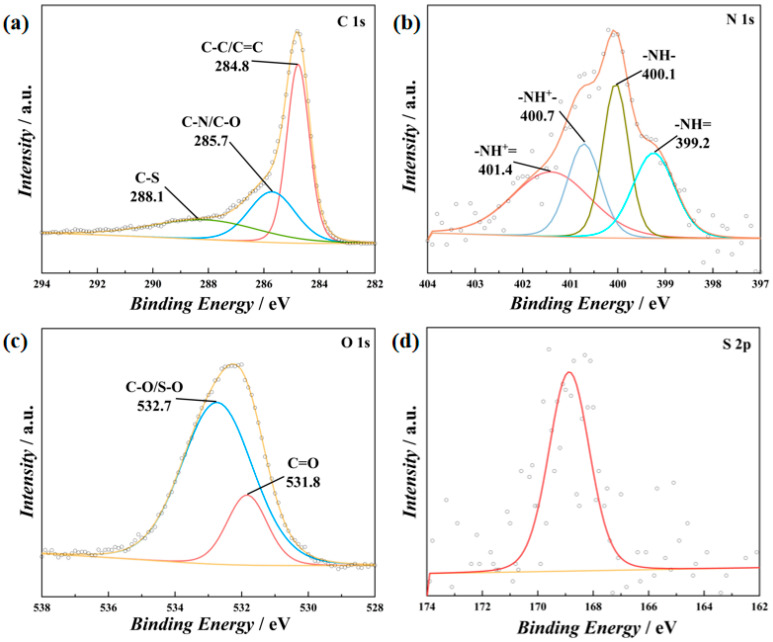
XPSs of (**a**) C 1s, (**b**) N 1s, (**c**) O 1s, and (**d**) S 2p regions of PT/CC-20.

Figure 3 displays the X-ray photoelectron spectra (XPS) of PT/CC-20. The fitted N 1s spectra of PT/CC-20 exhibit peaks at 401.4, 400.7, 400.1, and 399.2 eV, which correspond to protonated imine(–NH^+^=), protonated amine (–NH^+^–), amine (–NH–), and imine (–NH=) structures, respectively, in the PT/CC [29]. The doping level of the PT is acquired by calculating the percentage of protonated imino and amino groups over the overall N content to be 0.52. This value is slightly higher than the doping level of conducting polymers acquired by electropolymerization (0.33–0.50), which could be attributed to the oxidative potential applied during the electropolymerization process [19]. The O 1s spectra can be deconvoluted into peaks at 532.7 and 531.8 eV, which match the reported C–O or S–O and C=O groups, respectively. The presence of hydroxyl/ketone groups indicates that some s-triazine units of PT are oxidized. A weak S 2p peak is also found in the XPSs of PT/CC, which originates from the sulfate ions that are intercalated into the polymer matrix to balance the charge of the protonated amino or imino groups.

**Figure 4:**
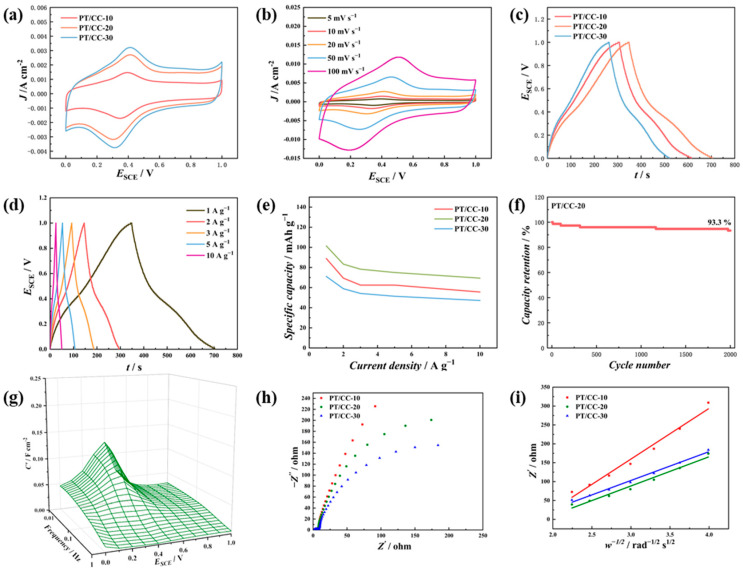
(**a**) CV of PT/CC at 20 mV $s^{-1}$ in 1 M $H_{2}SO_{4}.$ (**b**) CV of PT/CC-20 from 5 to 100 mV $s^{-1}$ in 1 M $H_{2}SO_{4}.$ (**c**) GCD of PT/CC at 1 A $g^{-1}$ in 1 M $H_{2}SO_{4}.$ (**d**) GCD of PT/CC-20 at the current densities from 1 to 10 A $g^{-1}$ in 1 M $H_{2}SO_{4}.$ (**e**) Specific capacities vs. GCD current densities of PT/CC in 1 M $H_{2}SO_{4}$. (**f**) The cycling stability of PT/CC-20 at 10 A $g^{-1}.$ The electrolyte is 1 M $H_{2}SO_{4}$. (**g**) Plots of C′ vs. f vs. E of PT/CC-20 in 1 M $H_{2}SO_{4}$. (**h**) The Nyquist plots of PT/CC in 1 M $H_{2}SO_{4}$. (**i**) The plot of Z′ vs. $\omega^{-1/2}$ of the PT/CC in 1 M $H_{2}SO_{4}$.

The charge storage performance of PT/CC in 1 M H_2_SO_4_ was then evaluated using a three-electrode system. Figure 4a shows the CV of PT/CC in 1 M $H_{2}{SO}_{4}$ at 20 mV $s^{-1}$. A pair of reversible redox peaks was observed for all the PT/CCs. The *E*_p/2_ (average of oxidation peak potential and reduction peak potential) for the PT/CCs were ~0.36 V_SCE_. This peak was not present in the CV of dissolved s-triazine in H_2_SO_4_ (Figure 1a, 1st cycle), which indicates that this redox process is related to the redox of polymer or to the functional groups formed during electropolymerization. As anodic potentials are applied during electropolymerization, radical formation followed by water attack is possible to generate hydroxyl/carbonyl structures in the polymer. Irreversible oxidation initiates at 1.0 V_SCE_, which could be the oxidation of PT or oxygen evolution. Therefore, the upper limit of the potential was selected as 1.0 V_SCE_. By integrating the charge under the cathodic half of CV and taking into consideration the mass of the PT at the electrode, the specific capacities of PT/CC-10, PT/CC-20, and PT/CC-30 were 96.8, 108.1, and 84.9 mAh $g^{-1}$, respectively. Figure 4b displays the CV of PT/CC-20 at scan rates ranging from 5 to 100 mV $s^{-1}$. As the scan rate increases, the separation of the redox peak potential increases. The log *i*_p_ (peak current) versus log *v* was plotted for the redox peak and is shown in Figure S2. The slopes of the oxidation peak and the reduction peak were 0.92 and 0.87, respectively, which indicates that both semi-infinite diffusion-controlled processes and a surface-capacitive controlled processes exist. Figure 4c shows the galvanostatic charge–discharge (GCD) curves of PT/CC at a current density of 1 A $g^{-1}$. Plateaus at 0.4 V were observed during both charging and discharging, which coincide with the redox peaks in CV, suggesting that the redox process contributes to the stored charge. The specific capacities of PT/CC-10, PT/CC-20, and PT/CC-30 calculated from the GCD were 86.1, 101.4, and 72.2 mAh $g^{-1}$, respectively.

The PT/CC was then evaluated at various GCD current densities (Figure 4d), and the specific capacities are summarized in Figure 4e. At all the current densities, PT/CC-20 exhibited the highest specific capacity, while the specific capacity of PT/CC-30 was the lowest. Also, at higher current densities, the specific capacities for all the PT/CCs decreased. This originates from the limited diffusion and redox kinetics at high current densities, which leads to a decreased number of electrochemically active sites for charge storage. When the current density varied from 1 to 10 A $g^{-1}$, the capacity retention of PT/CC-10, -20, and -30 was 62.5%, 68.4%, and 66.4%, respectively. The cycling stability of PT/CC-20 at 10 A $g^{-1}$ was evaluated using GCD (Figure 4f). After 2000 cycles at 10 A $g^{-1}$, the capacity retention of PT/CC-20 was 93.3% in 1 M $H_{2}{SO}_{4}$. Figure 4g displays the three-dimensional Bode plots (*C*′ vs. *f* vs. *E*) of PT/CC-20. This 3D Bode plot is reported to be used to identify the type of energy storage of the material [30]. The existence of a peak in the 3D Bode plot indicates that PT in aqueous acidic solution displays a battery-type behavior [31]. Figure 4h compares the EIS plots of the PT/CCs. The impedance spectra were acquired at 0 V_SCE_. A small semicircle in the high-frequency region represents the charge transfer resistance (*R*_CT_), while a slope represents the diffusion impedance [32]. Further analysis of the low-frequency data acquired from EIS by plotting *Z*′ versus *w*^−1/2^ is shown in Figure 4i. The slope (Warburg factor) can be correlated to the diffusion impedance [33,34]. For PT/CC-10, -20, and -30, the slopes were 134, 77, and 76 Ω s^−1/2^ rad^1/2^, respectively. PT/CC-30 exhibited the lowest slope, which indicates that the diffusion of ionic species at PT/CC-10 was the most facile.

**Figure 5:**
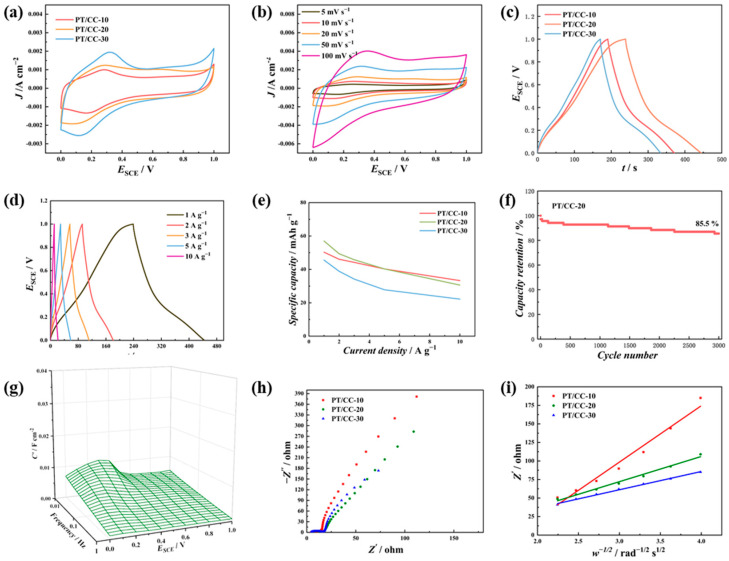
(**a**) CV of PT/CC at 20 mV $s^{-1}$ in 1 M ZnSO_4_. (**b**) CV of PT/CC-20 from 5 to 100 mV $s^{-1}$ in 1 M ZnSO_4_. (**c**) GCD of PT/CC at 1 A $g^{-1}$ in 1 M ZnSO_4_. (**d**) GCD of PT/CC-20 at the current densities from 1 to 10 A $g^{-1}$ in 1 M ZnSO_4_. (**e**) Specific capacities vs. GCD current densities of PT/CC in 1 M ZnSO_4_. (**f**) The cycling stability of PT/CC-20 at 10 A $g^{-1}$. The electrolyte is 1 M ${ZnSO}_{4}$. (**g**) Plots of C′ vs. f vs. E of PT/CC-20 in 1 M ${ZnSO}_{4}$. (**h**) The plot of -Z″ vs. Z′ of the PT/CC in 1 M ${ZnSO}_{4}$. (**i**) The plot of Z’ vs. $\omega^{-1/2}$ of the PT/CC in 1 M ZnSO_4_.

As the PT/CC exhibits the capability to store charge in sulfuric acid, it would be intriguing to see whether other types of ions can be stored by the PT/CC. Aqueous zinc ion storage is arousing increasing interests of researchers owing to its high safety and trivial assembly [35]. Therefore, the charge storage performance of the PT/CC was then evaluated in 1 M ZnSO_4_. Figure 5a displays the CV of the PT/CC in 1 M ZnSO_4_. All electrodes showed obvious redox peaks with *E*_p/2_ at ~0.22 V_SCE_. The pH of 1 M ZnSO_4_ is 4.3. The *E*_p/2_ shifts cathodically in 1 M ZnSO_4_ with a magnitude of 42 mV pH^−1^. This indicates that this redox process involves less than one proton transfer accompanied by one electron transfer. In 1 M ZnSO_4_, the [H^+^] is significantly lower, while the redox peak only slightly decreases in size. This indicates that the cationic species (Zn^2+^) can as well interact with these groups and participate in the redox processes. By integrating the cathodic area under the CV curves, the specific capacities of PT/CC-10, PT/CC-20, and PT/CC-30 were calculated to be 50.3, 57, and 45.6 mAh $g^{-1}$, respectively. Figure 5b illustrates the CV of PT/CC-20 at various scan rates. The redox peak potentials also exhibited a dependency on the scan rate (Figure S3), and the slope of log *i*_p_ versus log *v* was 0.74 and 0.78, respectively. This indicates that the redox processes in 1 M ZnSO_4_ also involve semi-infinite diffusion-controlled and surface-capacitive controlled processes. The galvanostatic charge–discharge (GCD) curves of PT/CC at 1 A g^−1^ are shown in Figure 5c. The specific capacities of PT/CC-10, PT/CC-20, and PT/CC-30 in 1 M ${ZnSO}_{4}$ were 67.3, 70.4, and 66.7 mAh $g^{-1}$, respectively. Figure 5d displays the GCD curves of PT/CC-20 at 1 to 10 A $g^{-1}$. When discharged at current densities of 1, 2, 3, 5, and 10 A $g^{-1}$, the specific capacities of PT/CC-20 were 57, 49.4, 45.8, 40.3, and 30.6 mAh $g^{-1}$, respectively. Figure 5e shows the variation in the specific capacities with different current densities for all the PT/CCs. The capacity retention at a GCD current density of 10 A $g^{-1}$ relative to 1 A $g^{-1}$ for PT/CC-10, PT/CC-20, and PT/CC-30 was 66.2%, 53.7%, and 48.7%, respectively. Figure 5f demonstrates the cycling stability of PT/CC-20 at 10 A $g^{-1}$, retaining 85.5% of its initial capacity after 3000 cycles. The 3D Bode plots also exhibited a peak at low frequency (Figure 5g), which implies that the PT/CC is a battery-type electrode material in 1 M ZnSO_4_. The impedance spectra of PT/CC-10, PT/CC-20, and PT/CC-30 are shown in Figure 5h. A larger *R*_CT_ than those in 1 M H_2_SO_4_ is shown, which suggests that the charge storage kinetics is more facile for the H^+^ than for the Zn^2+^. This is probably originated from the different sizes of the hydrated H^+^ and Zn^2+^, and their different dehydration behavior [36]. The diffusion impedance was also evaluated, and the values of the Warburg factors were 76, 34, and 24 Ω s^−1/2^ rad^1/2^ for PT/CC-10, -20, and -30, respectively. These values are lower than those in 1 M H_2_SO_4_, which indicates that the diffusion of active species in ZnSO_4_ is more facile than those in H_2_SO_4_.

In both electrolytes, PT/CC-20 exhibited the highest specific capacity. The origin of the high specific capacity lies in the combination of the abundant number of active sites per mass, facile charge storage kinetics, and faster diffusion process. For PT/CC-10, the small CV in both electrolytes indicates that the number of electrocatalytic active sites is limited, which is due to the insufficient amounts of polymers electrodeposited on CC [37]. In contrast, for PT/CC-30, a thick layer is formed, and the number of active sites per mass is limited, as some sites are covered and not accessible by ions in the electrolyte. Therefore, PT/CC-20 exhibited the best charge storage properties both in 1 M H_2_SO_4_ and in 1 M ZnSO_4_.

**Figure 6:**
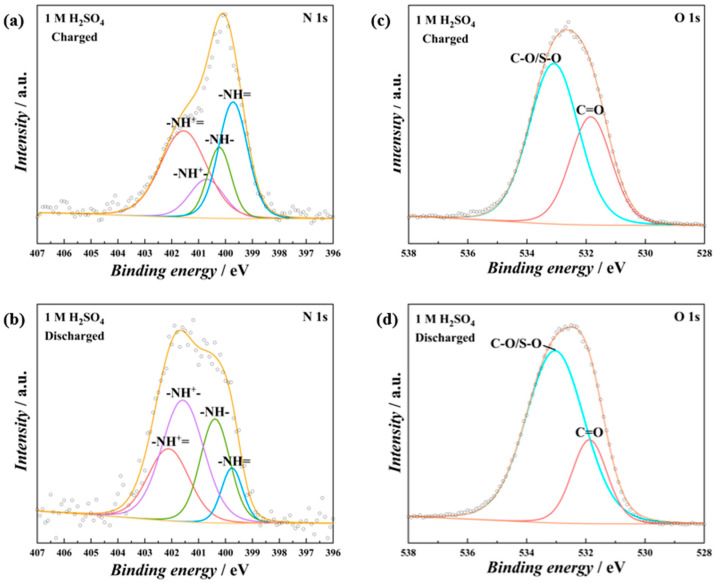
Deconvoluted XPSs of N 1s region of PT/CC-20 when (**a**) charged to 1.0 V and (**b**) discharged to 0 V in 1 M $H_{2}SO_{4}$. Deconvoluted XPSs of O 1s region of PT/CC-20 when (**c**) charged to 1.0 V and (**d**) discharged to 0 V in 1 M $H_{2}SO_{4}$.

**Figure 7:**
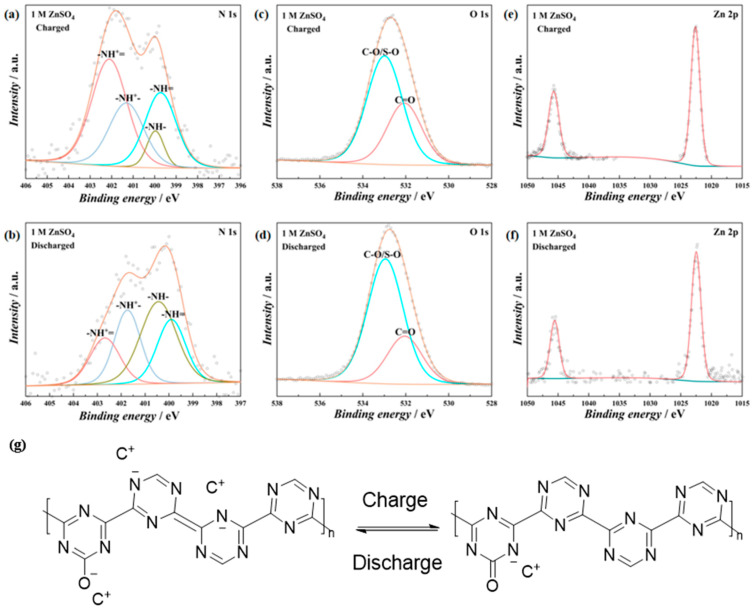
Deconvoluted XPSs of N 1s region of PT/CC-20 when (**a**) charged to 1.0 V and (**b**) discharged to 0 V in 1 M ${ZnSO}_{4}$. Deconvoluted XPSs of O 1s region of PT/CC-20 when (**c**) charged to 1.0 V and (**d**) discharged to 0 V in 1 M ${ZnSO}_{4}$. Deconvoluted XPSs of Zn 2p region of PT/CC-20 when (**e**) charged to 1.0 V and (**f**) discharged to 0 V in 1 M ${ZnSO}_{4}$. (**g**) Proposed charge storage mechanism of the PT/CC (C represents cation with unit charge).

As the PT/CC exhibited battery-type charge storage behavior, the charge storage mechanism was further investigated using XPSs at different states of charge and discharge (Figure 6). In 1 M H_2_SO_4_, at a charged state (Figure 6a), the imino structure (both -N= and -NH^+^=) accounts for 71.12% of the total N. While at the discharged state (Figure 6c), the imino structure reduces to 33.54%. This indicates that at the charged states, the amino groups are oxidized to imino groups [38], while the reverse process happens at the discharged state. For the O 1s spectra, at the charged state (Figure 6b), the carbonyl group (C=O) accounts for 35.93% of the total O, and at the discharged state (Figure 6d), this value reduces to 22.66%. This implies that the carbonyl/hydroxyl groups, which are probably generated during anodic electropolymerization, also participate in the redox process. In ZnSO_4_, a similar process happens, with the imino structure accounting for 68.28% at the charged state (Figure 7a), while at the discharged state, this value reduces to 39.71% (Figure 7b). A higher content of the carbonyl group was observed at the charged state (Figure 7c) than at the discharged state (Figure 7d). In addition, the content of Zn^2+^ increased at the discharged state compared to the charged state (Figure 7e,f). These results indicate that both the H^+^ and Zn^2+^ can be stored by the PT/CC. Therefore, the charge storage mechanism of the PT/CC is proposed in Figure 7g. Theoretical calculation indicates that the fully reduced state can be reached by 6 e^−^ reduction in one triazine ring [39,40]. However, the full reduction is barely possible in conducting polymers for charge storage. This proposed mechanism represents one possible unit in the PT structure. As the conducting polymer films formed by electropolymerization often involve a complex structure, this mechanism does not rule out other possibilities for charge storage. For example, besides the proposed redox process, the triazine ring can bind to cations (through cation–π interaction) and anions (anion–σ interaction), as well, to store charges.

**Figure 8:**
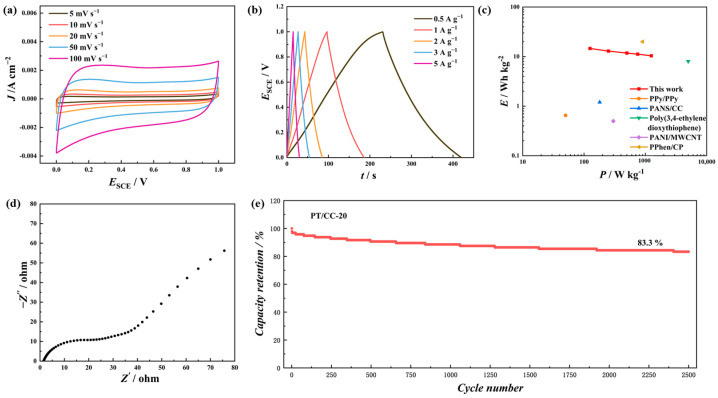
(**a**) CV of the device with 1 M $H_{2}{SO}_{4}$ aqueous electrolyte. (**b**) GCD profiles of the device at different current densities. (**c**) The Ragone plot comparing the device in this work with the literature systems based on conducting polymers. (**d**) The Nyquist plot. (**e**) The capacity retention versus cycle number of the device at 10 A $g^{-1}$.

In the three-electrode test, PT/CC-20 exhibited the highest specific capacity and stability. Therefore, a two-electrode test was conducted in 1 M $H_{2}{SO}_{4}$, with two PT/CC-20 as both positive and negative electrodes. Figure 8a displays the CV of the device from 5 to 100 mV $s^{-1}$. Different from the CV acquired using the three-electrode test, no obvious redox peaks appeared in the potential range (0 to 1.0 V), and the shape of the CV was rectangular. This indicates that no irreversible redox reactions occurred within the selected voltage window [41]. Figure 7b shows the GCD at various current densities. Triangular GCD curves are shown, which is typical for capacitive or peudocapacitive charge storage behavior. When the discharge current densities were 0.5, 1, 2, 3, and 5 A $g^{-1}$, the specific capacities were 105, 93, 85, 81, and 75 F $g^{-1}$, respectively. The Ragone plot of the device compared with the reported systems is shown in Figure 8c. At a current density of 1 A $g^{-1}$, the device’s energy density was 12.92 Wh ${kg}^{-1}$, and the power density was 250 W ${kg}^{-1}$. The EIS spectrum of this device at 0 V is shown in Figure 8d. The plot exhibits a semicircle at the high-frequency region and a slope line in the low-frequency region. These features are typical for the Nyquist plot of battery-type supercapacitors [42]. The stability test was carried out at a GCD current density of 10 A g^−1^. A high capacitance retention rate (83.3%) after 2500 repeated charge and discharge cycles was shown. This indicates that PT/CC is a promising electrode material for supercapacitors.

## 4. Conclusions

s-Triazine was, for the first time, electropolymerized onto a CC surface using electropolymerization in acidic aqueous solution. Both repetitive CV and galvanostatic methods can generate polymers at the CC surface. The PT exhibited a granular structure, with abundant pores and cracks. The charge storage properties of PT/CC at different electropolymerization durations were investigated in 1 M H_2_SO_4_ and in 1 M ZnSO_4_, with PT/CC-20 showing the highest specific capacity. A specific capacity of 101.4 mAh $g^{-1}$ at 1 A $g^{-1}$ in 1 M H_2_SO_4_ was achieved by PT/CC-20. In 1 M ZnSO_4_, a specific capacity of 50.3 mAh $g^{-1}$ was obtained at 1 A $g^{-1}.$ The PT exhibited battery-type behavior, and the good charge storage capacity resulted from a combination of an abundant number of active sites per mass, facile charge transport kinetics, and diffusion processes. The charge storage mechanism involves the redox of imino/amino groups and the carbonyl/hydroxyl groups, and the cations in solution interact with these groups. When assembled into a symmetric supercapacitor using two PT/CC-20s and electrolyte, an energy density of 12.92 Wh ${kg}^{-1}$ and a power density of 250 W ${kg}^{-1}$ at 1 A g^−1^ were achieved. After 2500 cycles at 10 A g^−1^, 83.3% capacitance retention was maintained. This study demonstrates that a new conducting polymer, PT, can be easily prepared by electropolymerization and is a promising electrode material for charge storage in acidic aqueous solution. Though the strong hydrophobicity of the triazine unit may interfere with the charge storage performance in aqueous media, tuning the pH of the electrolyte to protonate the triazine unit, coordination with ions in the electrolyte and fabrication of PT films with a high specific surface area are possible routes to achieve a high specific capacity. Given the capability of PT to store Zn^2+^ in 1 M ZnSO_4_, we envisage that it can also be a good candidate as the electrode material for an aqueous zinc ion battery.

## Data Availability

The original contributions presented in the study are included in the article/Supplementary Materials; further inquiries can be directed to the corresponding author.

## Supplementary Materials

The following supporting information can be downloaded at https://www.mdpi.com/article/10.3390/polym16233266/s1: Figure S1: *E*–*t* curve for electropolymerization of 5 mM triazine in 1 M H_2_SO_4_ at 0.01 A cm^−2^. The working electrode is CC [43,44,45]; Figure S2: CV and log (*i*, mA) versus log (*v*, mV s^−1^) plots at peak currents in 1 M H_2_SO_4_; Figure S3: CV and log (*i*, mA) versus log (*v*, mV s^−1^) plots at specific peak currents in 1 M ZnSO_4_; Figure S4: Bode plot of symmetric supercapacitor with 1 M H_2_SO_4_ aqueous electrolyte; Figure S5: Plots of C′ and C″ vs. *f* of the symmetric supercapacitor with 1 M H_2_SO_4_ aqueous electrolyte [8,46]; Figure S6: The SEM images of the PT/CC-10 (left) and 30 (right); Figure S7: Proposed redox processes of the s-triazine in 1 M H_2_SO_4_. Table S1. Components of the deconvoluted C 1s XPS spectra of the PT/CC-20 in 1 M H_2_SO_4_. Table S2. Components of the deconvoluted N 1s XPS spectra of the PT/CC-20 in 1 M H_2_SO_4_. Table S3. Components of the deconvoluted O 1s XPS spectra of the PT/CC-20 in 1 M H_2_SO_4_. Table S4. Components of the deconvoluted N 1s XPS spectra of the PT/CC-20 charged or discharged in different solutions. Table S5. Components of the deconvoluted O 1s XPS spectra of the PT/CC-20 charged or discharged in different solutions. Table S6. Comparison of specific capacity and cyclic stability among some previously reported polymer based electrochemical energy storage systems.
