# Supplementary material for: Electropolymerization of s-Triazines and Their Charge Storage Performance in Aqueous Acidic Electrolytes
Source: Polymers (Basel). 2024 Nov 24;16(23):3266. doi: 10.3390/polym16233266 (PMC11644275; doi:10.3390/polym16233266)
Supplement: Supplementary file 1 [file polymers-16-03266-s001.zip › polymers-3234406-supplementary.pdf]

Supporting Information

Electropolymerization of s-triazine and their charge storage performance in aqueous acidic electrolytes

Shaotong Pei[a,], Bo Lan[a], Xueting Bai[a], Yunpeng Liu[a], Xinyang Li[a], Chao Wang[b,*]*

a. *Hebei Provincial Key Laboratory of Power Transmission Equipment Security Defense, North China Electric Power University, Baoding, Hebei 071003, China*

b. *Department of Chemistry and Chemical Engineering, Shaanxi University of Science and Technology, Xi'an, Shaanxi 710021, China*

* Corresponding authors:

peishaotong@ncepu.edu.cn

cwang@sust.edu.cn

Table of Content

1. **Experimental**
2. **Electrochemistry**
3. **SEM**
4. **Tables**
5. **Capacity comparison**

1. Experimental

Chemicals

The following reagents were used without further purifications. 1,3,5-triazine ($C_3H_3N_3$, AR, 97%, Shanghai BiDe Pharmaceutical Technology Co., Ltd.), concentrated sulfuric acid (H_2SO_4, AR, 98.0%, Shanghai HaoHong Bio-Pharmaceutical Technology Co., Ltd.), potassium ferricyanide ($K_3[Fe(CN)_6]$, AR, 99.5%, Tianjin RuiJinTe Chemicals Co., Ltd.), potassium chloride (KCl, AR, 99.5%, FuChen (Tianjin) Chemical Reagents Co., Ltd.), zinc sulfate ($ZnSO_4$, AR, 99.8%, Shanghai HaoHong Bio-Pharmaceutical Technology Co., Ltd.), carbon cloth (SCC130, Suzhou ShengErNuo Technology Co., Ltd.) and doubly distilled water.

Instrumentation

X-ray photoelectron spectroscopy (XPS) was carried out using a Shimadzu/Krayos AXIS Ultra DLD at room temperature and ultra-high vacuum (UHV) conditions. The survey spectra were acquired in the binding energy range of 0 – 1200 eV with a step energy of 0.7 eV, a pass energy of 160 eV, and a sweep time of 180 s. The high-resolution spectra were obtained with an energy envelope of 30 eV, a pass energy of 20 eV, a step size of 0.05 eV, and a sweep time of 180 s. No charge correction is applied during the XPS analysis. All the high-resolution XPS spectra were calibrated against the C 1 s peak at 284.6 eV when analyzed using CasaXPS software. X-ray diffraction (XRD) was

performed on X-ray powder diffractometer (Rigaku SmartLab SE, Japan) equipped with a Cu target (λ = 0.154 nm). Field emission scanning electron microscope (TESCAN MIRA LMS, The Czech Republic) equipped with electron diffraction spectroscope was used to observe the morphology and elemental distribution of the film.

Electrochemical measurements

The electrochemical measurements are carried out at room temperature using CHI760E electrochemical workstation, and EIS was recorded in the frequency range of $10^6 \sim 0.01$ Hz under potential amplitude 5 mV. The specific capacity (C,mAh g^{-1}) was calculated from the discharge part of the galvanostatic charge-discharge (GCD) curve using Equation (S1).

$$C_{S,GCD} = \frac{It}{m} \quad \text{(S1)}$$

Where I(mA) is the discharge current, t(h) is the discharge time, and m(g) is the mass of the active material on the electrode.

The specific capacity (C,mAh g^{-1}) was also calculated from the integrated charge (Q,C) obtained by cyclic voltammetry (CV) scans using Equation (S2).

$$C_{S,CV} = \frac{Q}{3.6m} \quad \text{(S2)}$$

The capacitance of the PT/CC two-electrode energy storage system was calculated from the GCD curve using Equation (S3).

$$C_{cell} = \frac{It}{2m \times \Delta V} \quad \text{(S3)}$$

Where C_{cell} (F g^{-1}) is the specific capacity based on the mass of electrochemically active material, I is the current in A, and $\Delta V(V)$ is the potential window.

The energy density and power density of the solid-state supercapacitor were calculated using Equations (S4) and (S5).

$$E = \frac{1}{2\times 3.6\times 2m}\int_0^t V dt \qquad \text{(S4)}$$

$$P = \frac{E\times 3600}{t} \qquad \text{(S5)}$$

where E (Wh kg^{-1}) and P (W kg^{-1}) correspond to energy density and power density, respectively.

2. Electrochemistry

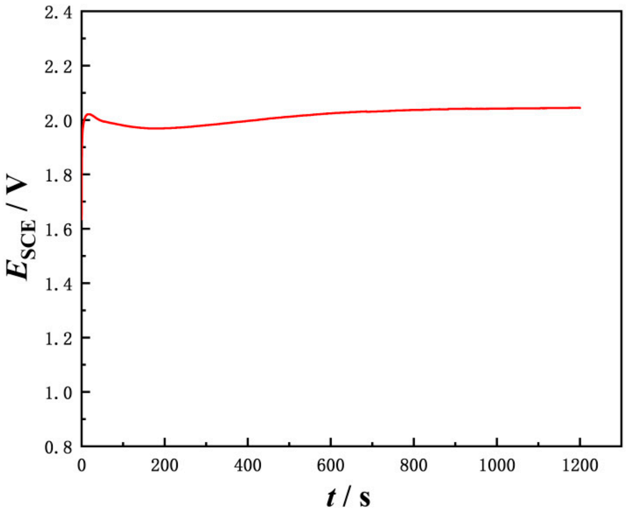

Figure S1. *E* - *t* curve for electropolymerization of 5 mM triazine in 1 M H_2SO_4 at 0.01 A cm^{-2}. The working electrode is CC.

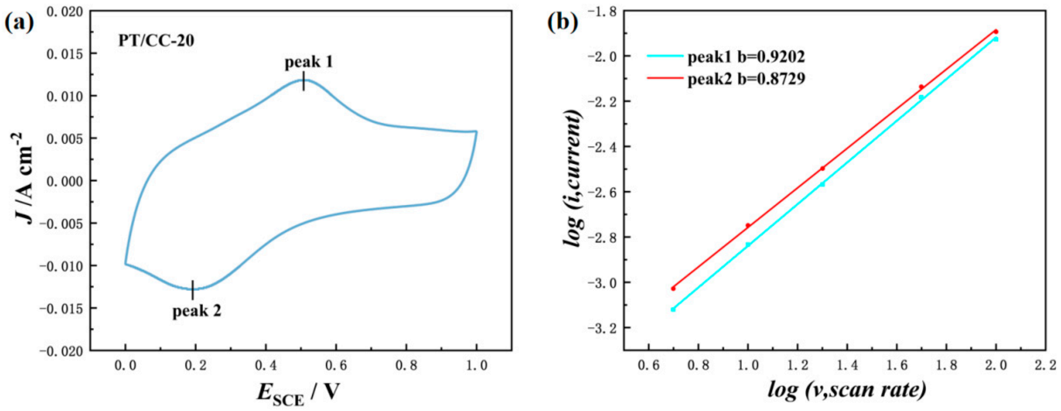

Figure S2. CV and log (*i*, mA) versus log (*v*, mV s^{-1}) plots at peak currents in 1 M H_2SO_4.

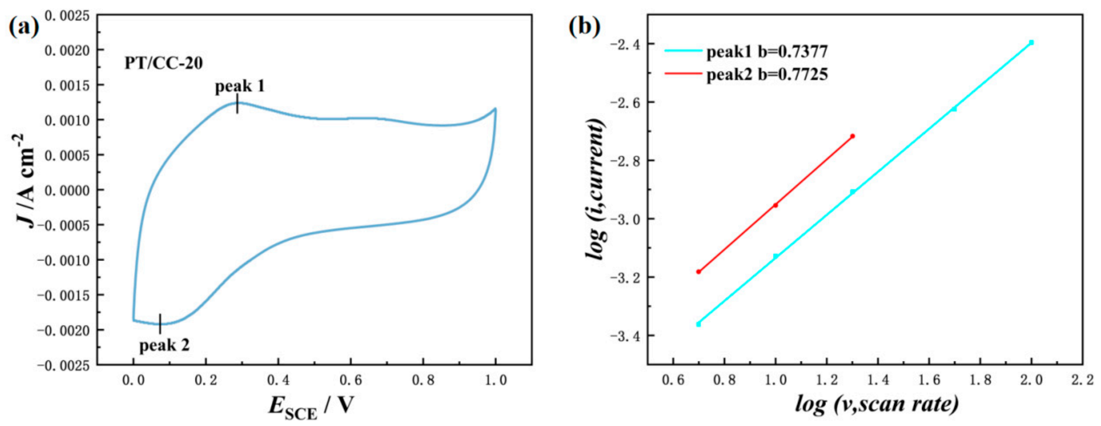

Figure S3. CV and log (i, mA) versus log (v, mV s^{-1}) plots at specific peak currents in 1 M $ZnSO_4$.

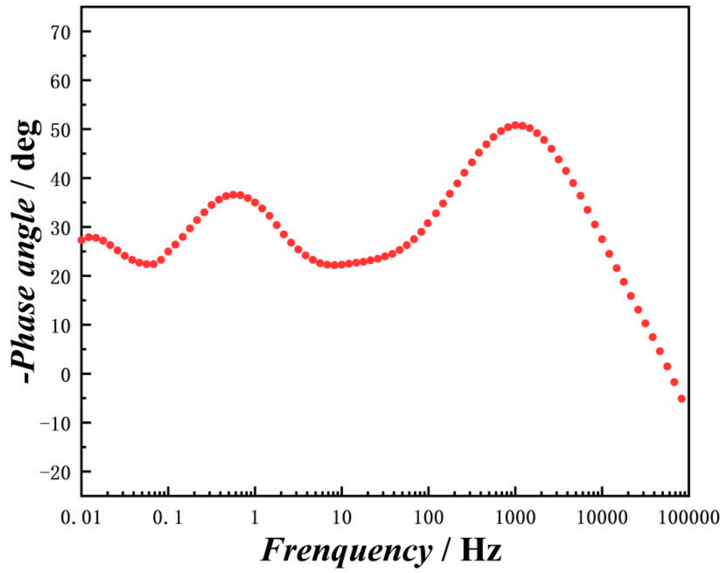

Figure S4. Bode plot of symmetric supercapacitor with 1 M H_2SO_4 aqueous electrolyte.

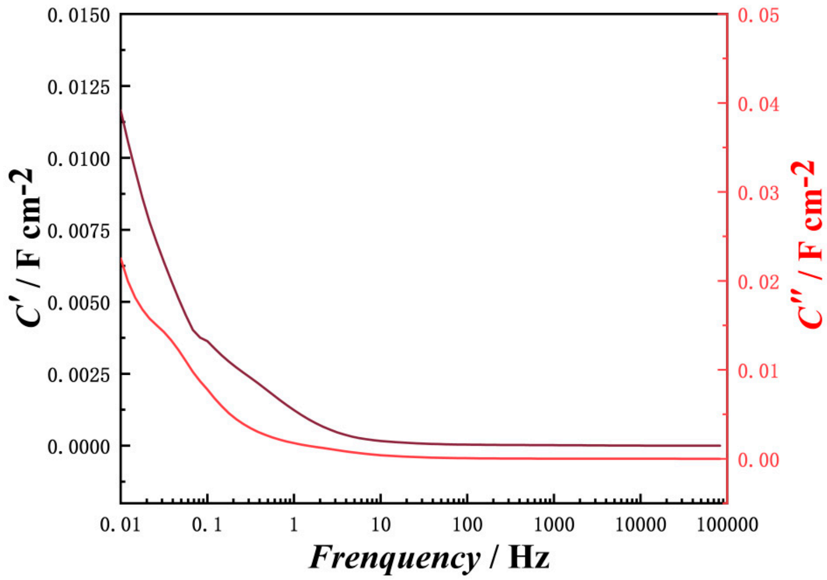

Figure S5. Plots of *C′* and *C′′ vs. f* of the symmetric supercapacitor with 1 M H_2SO_4 aqueous electrolyte.

3. SEM

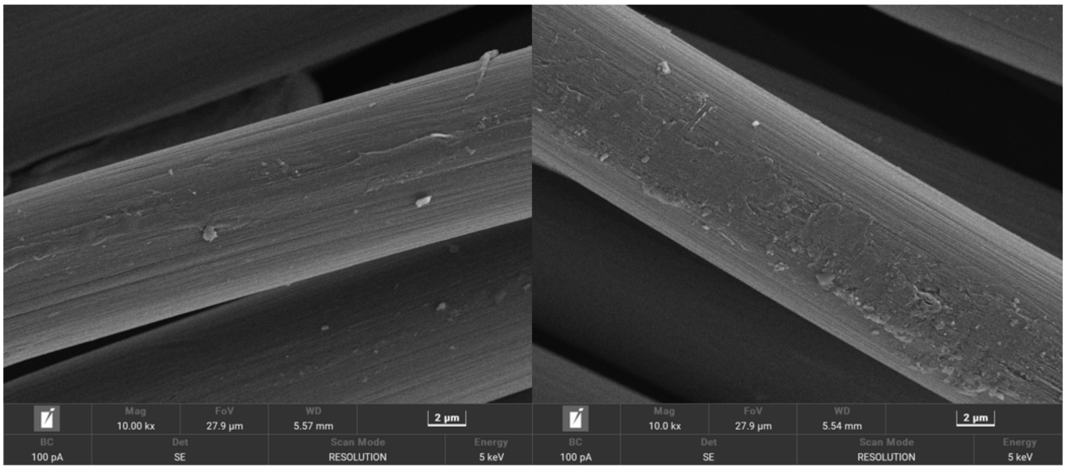

Figure S6. The SEM images of the PT/CC-10 (left) and 30 (right).

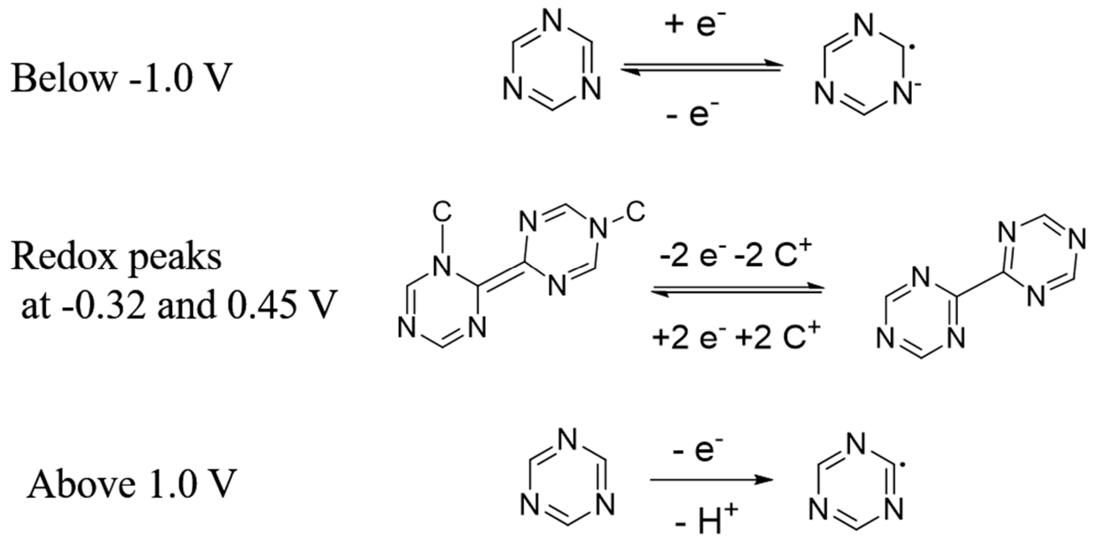

Figure S7. Proposed redox processes of the s-triazine in 1 M H_2SO_4.

4. Tables

Table S1. Components of the deconvoluted C 1s XPS spectra of the PT/CC-20 in 1 M H_2SO_4.

	284.8 eV<br>C=C/C-C	285.7 eV<br>C-N/C-O	288.1 eV<br>C-S
1 M H_2SO_4	47.24	27.79	24.97

Table S2. Components of the deconvoluted N 1s XPS spectra of the PT/CC-20 in 1 M H_2SO_4.

	399.2 eV<br>-NH=	400.1 eV<br>-NH-	400.7 eV<br>$-NH^{+}-$	401.4 eV<br>$-NH^{+}=$
1 M H_2SO_4	22.78	25.20	19.76	32.26

Table S3. Components of the deconvoluted O 1s XPS spectra of the PT/CC-20 in 1 M H_2SO_4.

	531.8 eV<br>C=O	532.7 eV<br>C-O/S-O
1 M H_2SO_4	19.70	80.30

Table S4. Components of the deconvoluted N 1s XPS spectra of the PT/CC-20 charged or discharged in different solutions.

	399.2 eV -NH=	400.1 eV -NH-	400.7 eV $-NH^{+}-$	401.4 eV $-NH^{+}=$
1 M H_2SO_4 Charged	32.66	16.14	12.75	38.46
1 M H_2SO_4 Discharged	9.50	25.05	41.41	24.04
1 M $ZnSO_4$ Charged	26.08	6.99	24.73	42.20
1 M $ZnSO_4$ Discharged	22.43	36.45	23.85	17.28

Table S5. Components of the deconvoluted O 1s XPS spectra of the PT/CC-20 charged or discharged in different solutions..

	531.8 eV C=O	532.7 eV C-O/S-O
1 M H_2SO_4 Charged	35.93	64.07
1 M H_2SO_4 Discharged	22.66	77.34
1 M $ZnSO_4$ Charged	35.79	64.21
1 M $ZnSO_4$ Discharged	27.45	72.55

5. Capacity Comparison

Table S6. Comparison of specific capacity and cyclic stability among some previously reported polymer based electrochemical energy storage systems.

Material	Electrolyte	Specific capacity	Cyclic stability	Ref.
PT/CC	1 M H_2SO_4	93 F g^{-1} at 1 A g^{-1}	93.3% after 2000 cycles	This work
PPy	PVA/H_2SO_4	23.2 F g^{-1} at 0.5 A g^{-1}	81.0% after 1000 cycles	[1]
PANS/CC	1 M H_2SO_4	307.8 C g^{-1} at 1 A g^{-1}	79.9% after 2500 cycles	[2]
Poly(3,4-ethylenedioxythiophene)	1 M H_2SO_4	86.81 F g^{-1} at 1 mA cm^{-2}	71.6% after 1000 cycles	[3]
PANI/MWCNT	PVA/H_2SO_4	180 F g^{-1} at 0.5 A g^{-1}	85.0% after 1500 cycles	[4]
PPhen/CP	1 M H_2SO_4	401.4 C g^{-1} at 1 A g^{-1}	81.6% after 2000 cycles	[5]
	1 M KOH	349.6 C g^{-1} at 1 A g^{-1}	75.43% after 2000 cycles	

References

[1] H. Zhuo, Y. Hu, Z. Chen, L. Zhong, Carbohydr. Polym. 215 (2019) 322–329.

[2] Y. Tian, Y. Yang, Y. Wu, Z. Zhou, Y. Li, J. Wang, S. Liu, C. Wang, J. Energy Storage 72 (2023) 108308.

[3] T.-R. Lv, W.-H. Zhang, Y.-Q. Yang, J.-C. Zhang, M.-J. Yin, Z. Yin, K.-T. Yong, Q.-F. An, Small 19 (2023) 2301071.

[4] M.Z. Khan, I.H. Gul, M.M. Baig, M.A. Akram, Electrochimica Acta 441 (2023) 141816.

[5] C. Wang, Z. Zhou, Q. Tian, X. Cao, Y. Wu, S. Liu, J. Wang, Chem. Eng. J. 433 (2022) 134483.
